# The Impact of Pandemics Sentiment on the Global Art Market

**DOI:** 10.3389/fpubh.2021.799658

**Published:** 2021-11-29

**Authors:** Wei Wang

**Affiliations:** College of Economics and Management, Nanjing University of Aeronautics and Astronautics, Nanjing, China

**Keywords:** the COVID-19, global art markets, hedging purposes, quantile regression estimations, pandemics sentiment

## Abstract

This paper investigates the effects of pandemics sentiment (the World Pandemics Discussion Index) on the returns of the global art market from 1998Q1 to 2021Q2 at the global level. The Ordinary Least Squares and the Quantile Regression estimations indicate that global pandemics sentiment positively affects the returns of the global art market. This evidence means that investing in the art market can hedge the uncertainty shocks related to pandemics at the global level.

## Introduction

The COVID-19 pandemic has significantly affected all aspects of economics and social life since the first quarter of 2020. The pandemic has rolled out many public health problems and has been the leading issue in the twenty-first century (1). According to Hasell et al. (2), the COVID-19 pandemic has caused the death of 4.5 Million people from January 2020 to July 2021. At this juncture, economic, financial, and social indicators have also been affected by the COVID-19 pandemic. Baker et al. (3, 4) indicate that uncertainty related to the COVID-19 has been the great source of uncertainty that the modern economies have exposed.

It is important to note that fiscal stimulus packages and expansionary monetary policy implications during the COVID-19 pandemic have created higher inflation risks. Therefore, investors seek to diversify their portfolios. At this stage, art materials can also be an alternative instrument to diversify portfolios or hedge against inflation risk. Therefore, the global art market is expected to grow from $347B in 2020 to $405B in 2021 (5).

Indeed, previous papers have shown that art pieces (especially paintings) have been categorized as investment instruments [see, e.g., (6–27)]. These papers have indicated that arts are using an alternative investment to traditional financial assets to diversify portfolios, or art investments can be used for hedging against the inflation risk at the global or the country levels.

Furthermore, during the COVID-19 pandemic, policymakers have implemented lockdowns and closures of public areas, including art galleries shopping centers (28). Social distancing measures have also negatively affected the interactions among the people (29). Meanwhile, various international activities have been limited during the COVID-19 pandemic (30). These modern lifestyle changes could have also affected the way of behaving, including purchasing and selling decisions. On the one hand, the art markets also experienced various changes and uncertainties during the COVID-19 pandemic. Art galleries and auction houses are typically the leading venues in the art markets, and auctions lead to an efficient price level in art markets (31, 32). However, restrictions on meetings and international travel restrictions have significantly affected the size of art events in the art galleries and auction houses.

On the other hand, online auction platforms have enhanced, increasing the interest in art pieces. Various art galleries and museums have opened up online view options, and lots of people can visit the convention center without payment. Auction houses have increased their infrastructure and online platforms for promoting art pieces.

Given these backdrops, we investigate the effects of pandemic sentiment on the global art market. As we have discussed, there can be a negative or a positive impact of pandemics on the global art market. It is important to note that our data capture the quarterly sample from 1998Q1 to 2021Q2, including the COVID-19 pandemic (2020Q1–2021Q2). Besides, the sample includes other pandemics, such as Avian Flu, Bird Flu, Ebola, Middle East Respiratory Syndrome (MERS), Severe Acute Respiratory Syndrome (SARS), and Swine Flu. Even though most of the pandemics have remained regional, they can also affect the global art market due to their significant impact on regions like China and the Middle East. These regions also have an increasing interest in investing in art materials.

To the best of our knowledge, this is the first paper in the literature to analyze the effects of pandemics sentiment on the returns of the global art market. For this purpose, we utilize the Ordinary Least Squares (OLS) and the Quantile Regression estimations and find that global pandemics sentiment positively affects the returns of the global art market. Our findings indicate that investing in art markets can be used to hedge against the uncertainty shocks related to global pandemics.

The remaining parts of the paper are structured as follows. Section Data and Methodology includes the data details and explains the econometric methodology, such as the unit root tests and the OLS and Quantile Regression estimations. Section Empirical Findings discusses the empirical findings, and Section Concluding Remarks presents the concluding remarks.

## Data and Methodology

### Data

This paper focuses on the relationship between two indicators: Global Arts Market (*Global_USD*) and Global Pandemics Sentiment (*GLOBAL_PANDEMICS*). The paper uses the quarterly frequency data from 1998Q1 to 2021Q2. Thus, we have 94 observations, which are suitable for time-series analysis.

The global art market is measured by the “global art market” index (1998Q1 = 100), which is based on the real prices of the United States Dollar (USD). The related data are obtained from artprice.com. Note that Artprice.com calculates the global art market index based on the prices of more than 30 million materials. The global price index also covers more than 700,000 artists. The price data comes from 6,300 auction houses all over the World (33).

Global pandemics sentiment is measured by the World Pandemics Discussion Index (WPDI), and the related data are obtained from https://worlduncertaintyindex.com/data/ introduced by Ahir et al. (34). The WPDI measure is based on searching for words related to the discussion of pandemics in the Economist Intelligence country reports, including Severe Acute Respiratory Syndrome, SARS, Avian Flu, H5N1, Swine Flu, H1N1, Middle East Respiratory Syndrome, MERS, Bird Flu, Ebola, Coronavirus, COVID-19, Influenza, H1V1, World Health Organization, and WHO. A higher level of the WPDI indicates a higher pandemics sentiment.

We also consider the logarithmic returns of the global art price index and the global pandemics sentiment following the unit root test results. We report the level and the returns of the global art price index and the global pandemics sentiment in Table 1. There are positive returns of the global art market on average, and the change in global pandemics sentiment is positive during the period under concern.

**Table 1:** Descriptive summary statistics (1998Q1–2021Q2).

**INDICATOR**	**GLOBAL_PANDEMICS**	**GLOBAL_USD**	**PANDEMICS_RETURN**	**ART_RETURN**
Mean	21.83542	154.8021	0.034545	0.006118
Median	1.699421	150.7463	−0.066987	0.009806
Max.	416.3459	243.2836	3.945514	0.358945
Min.	0.061256	95.52239	−1.641465	−0.442644
Std. Dev.	73.63242	38.37482	0.913938	0.141998
Skewness	3.98757	0.466349	1.413463	−0.086829
Kurtosis	18.06275	2.360128	7.664214	3.160983
Observations	94	94	93	93

In Table 2, we also provide the correlation matrix between the global art price index and the global pandemics sentiment. We observe a positive correlation (0.19) between the returns of the global art market and the global pandemics sentiment, and the correlation is statistically significant at the 5% level.

**Table 2:** Correlation matrix (1998Q1–2021Q2).

**Indicator**	**PANDEMICS_RETURN**	**ART_RETURN**
PANDEMICS_RETURN	1	–
ART_RETURN	0.1902	1
Probability	(0.0393)	–

### Econometric Methodology

We start with utilizing the unit root test procedures. For this purpose, we run the Augmented Dickey-Fuller (ADF) unit root test of Dickey and Fuller (35, 36) from 1998Q1 to 2021Q2. The ADF test results are based on (i) constant term, (ii) trend term, and (iii) both constant and trend terms. The results are based on the global art market's level and returns and the global pandemics sentiment. We provide the one-sided *p*-values calculated by MacKinnon (37).

It is also important to note that the OLS regression measures the impact of a one per cent change in the global pandemics sentiment on the mean of the returns of the global art market. In other words, the OLS regressions estimate the conditional mean of the returns of the global art market, given the value of the global pandemics sentiment. At this stage, we are also interested in analyzing the effects on certain quantiles (e.g., the median, the 90th percentile, and the 10th percentile) of the distribution of the returns of the global art market instead of the mean. At this stage, the statistical properties of the conditional quantiles are significantly different from that of the conditional mean. Therefore, the classical OLS technique breaks down in the quantile regressions. At this point, we implement the Quantile regression in STATA by using the *qreg* command. Therefore, we estimate the following model:


(1) $$\begin{array}{r} Global\text{\_}USD_{t}=\alpha_{0}+\alpha_{1}GLOBAL\text{\_}PANDEMICS_{t}+\mu_{t} \end{array}$$

Where *Global*_*USD*_*t*_ is the index of Global Arts Market, *GLOBAL*_*PANDEMICS*_*t*_ is the Global Pandemics Sentiment measured by the WPDI, and is the error term.

## Empirical Findings

Table 3 reports the results of the ADF unit root tests from 1998Q1 to 2021Q2. The ADF results are based on the level and the first differences of the series. The results are based on the model of (i) the constant term, (ii) the trend term, and (iii) both constant and trend terms. MacKinnon (37) one-sided *p*-values are reported. The global art price index follows a unit root process at the level series in all three models. In addition, the global pandemics sentiment is stationary. Therefore, we consider the returns of the global art price index and the global pandemics sentiment to capture the stationarity of the series during the estimations. We confirm that both the returns and the first differences of the series are stationary.

**Table 3:** Results of the ADF unit root tests (1998Q1–2021Q2).

		**GLOBAL_USD**	**GLOBAL_PANDEMICS**	**ART_RETURN**	**PANDEMICS_RETURN**
**At level**
With constant	*t*-statistic	−1.8673	−3.1658	−8.1635	−8.7746
	Prob.	0.3462	0.0254	0	0
		n0	^**^	^***^	^***^
With trend	*t*-statistic	−1.4909	−3.5658	−8.356	−8.7917
	Prob.	0.8257	0.0386	0	0
		n0	^**^	^***^	^***^
With constant and trend	*t*-statistic	−0.0577	−2.9592	−8.1885	−8.7866
	Prob.	0.6609	0.0035	0	0
		n0	^***^	^***^	^***^
		**d(GLOBAL_USD)**	**d(GLOBAL_PANDEMICS)**	**d(ART_RETURN)**	**d(PANDEMICS_RETURN)**
**At first difference**
With constant	*t*-statistic	−8.249	−5.9151	−6.0925	−9.3351
	Prob.	0	0	0	0
		^***^	^***^	^***^	^***^
With trend	*t*-statistic	−8.4064	−6.203	−6.0473	−9.2739
	Prob.	0	0	0	0
		^***^	^***^	^***^	^***^
With constant and trend	*t*-statistic	−8.2838	−5.8311	−6.1415	−9.3959
	Prob.	0	0	0	0
		^***^	^***^	^***^	^***^

*** *p < 0.01 and*

** *p < 0.05; (n0): not significant*.

* *MacKinnon (37) one-sided p-values*.

Furthermore, Table 4 provides the findings of the OLS and the Quantile Regressions estimations from 1998Q1 to 2021Q2. It is observed that the global pandemics sentiment positively affects the global art returns at all quantiles and the OLS estimations. According to the results of the OLS estimations, there is a positive impact of the global pandemics sentiment on global art returns. The coefficient is 0.031 in the OLS estimations, and it is statistically significant at the 5% level. The results of the Quantile Regressions also show the positive effects of the global pandemics sentiment on the global art returns in every quantile. However, the coefficients are only statistically significant for the quantiles from 10 to 50%. According to the results of the quantiles from 60 to 90%, the coefficients are statistically insignificant. This evidence means that a higher jump in the global pandemics sentiment does not significantly affect the global art returns.

**Table 4:** Results of the OLS and the quantile regression estimations of global art returns (1998Q1–2021Q2).

	**(1)**	**(2)**	**(3)**	**(4)**	**(5)**	**(6)**	**(7)**	**(8)**	**(9)**	**(10)**
	**OLS**	**10%**	**20%**	**30%**	**40%**	**50%**	**60%**	**70%**	**80%**	**90%**
d(GLOBAL_PANDEMICS)	0.031^**^	0.019^**^	0.041^*^	0.051^*^	0.038^**^	0.026^***^	0.017	0.015	0.026	0.035
Constant	0.003	−0.174^***^	−0.111^***^	−0.087^***^	−0.039^*^	0.003	0.037	0.094^***^	0.115^***^	0.171^***^
Observations	93	93	93	93	93	93	93	93	93	93

*** *p < 0.01*,

** *p < 0.05, and*

* *p < 0.1*.

## Concluding Remarks

In this paper, we analyse the effects of global pandemics sentiment (the World Pandemics Discussion Index) on the returns of the global art market from 1998Q1 to 2021Q2. We utilize the OLS and the Quantile Regression estimations. We find that global pandemics sentiment has a positive impact on the returns of the global art market. This evidence means that investing in art markets can be used for hedging against the uncertainty shocks related to global pandemics.

However, it is important to note that our evidence is limited to the global art market and pandemic sentiment. There are also available datasets for the advanced arts markets, such as France, the United Kingdom, and the United States. Therefore, future papers can focus on the country cases with the sub-indices of art markets data. Future papers can also utilize new methods, such as the Quantile Coherency measure provided by Barunik and Kley (38). In so doing, future papers can measure the dependency among pandemics sentiment, financial markets, and art markets.

## Data Availability Statement

Publicly available datasets were analyzed in this study. This data can be found here: worlduncertaintyindex.com/data/; https://www.artprice.com.

## Author Contributions

The author confirms being the sole contributor of this work and has approved it for publication.

## Conflict of Interest

The author declares that the research was conducted in the absence of any commercial or financial relationships that could be construed as a potential conflict of interest.

## Publisher's Note

All claims expressed in this article are solely those of the authors and do not necessarily represent those of their affiliated organizations, or those of the publisher, the editors and the reviewers. Any product that may be evaluated in this article, or claim that may be made by its manufacturer, is not guaranteed or endorsed by the publisher.
